# Supplementary material for: Self-reorganization and Information Transfer in Massive Schools of Fish
Source: ArXiv. 2025 Jun 3:arXiv:2505.05822v2. Originally published 2025 May 9. Preprint. [Version 2] (PMC12083704)
Supplement: Supplement 1 [file NIHPP2505.05822v2-supplement-1.pdf]

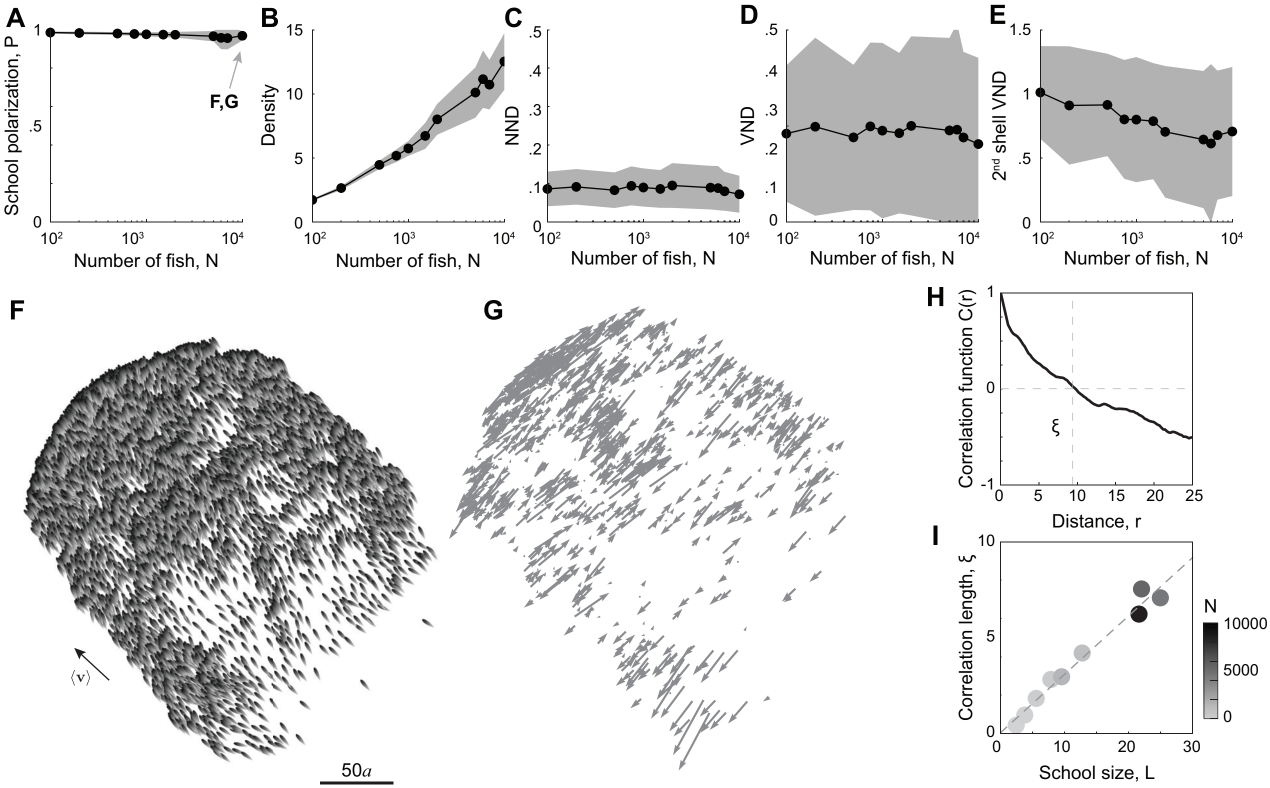

Figure S1: **Polarized schools do not split in the absence of hydrodynamic interactions. A.** Average polar order parameter P is nearly unchanged with increasing number of swimmers N when $I_f = 0$. **B.** density increases monotonically with increasing number of swimmers. **C.** average nearest neighbor and **D.** average distance to Voronoi neighbors are nearly unchanged with increasing number of swimmers, albeit with larger fluctuations in the latter. **E.** average distance to second shell Voronoi neighbors decreases with increasing number of swimmers. Snapshots of **F.** the school composed of $10,000$ swimmers and **G.** corresponding velocity fluctuations in the absence of hydrodynamic interactions. **H.** Correlation function plotted as a function of distance for the snapshots in **F.** and**G.**. **I.** Correlation length ξ is a linear function of school size L. The slope of the fitting line is 0.30. The slope is close to the slope we got with hydro (Fig. 4D) and in [12]. In all simulations, total integration time is $T = 1000$.

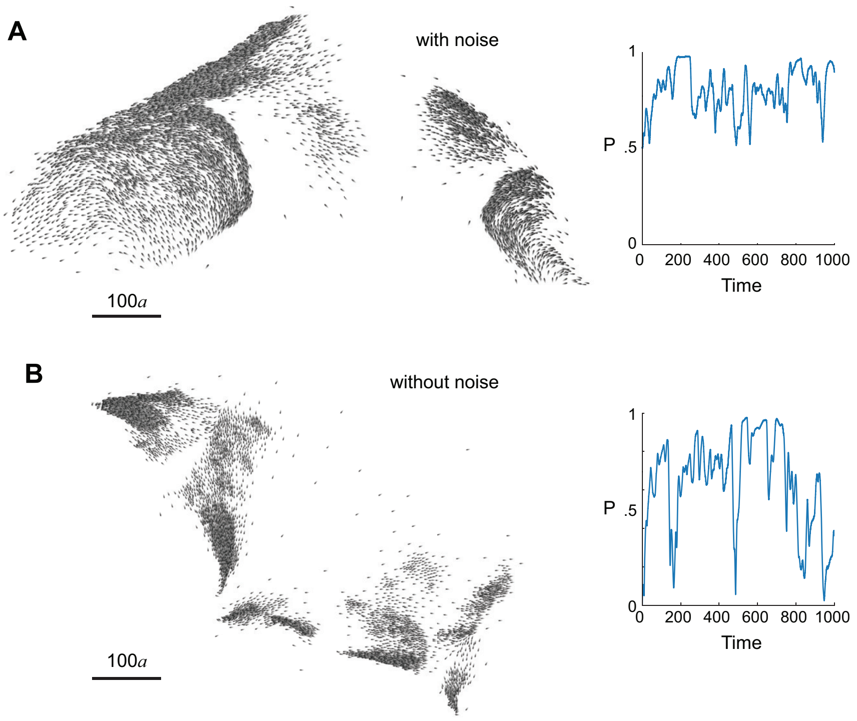

Figure S2: **Noise is not necessary for self-organization. A.** A snapshot and time evolution of polar order parameter P for a case with $N = 10,000$ swimmer with noise ($I_n = 0.5$). **B.** A snapshot and time evolution of polar order parameter P for a case with $N = 10,000$ swimmer without noise ($I_n = 0$). Parameter values: $N = 10,000$, $I_a = 9$, and $I_f = 0.01$.

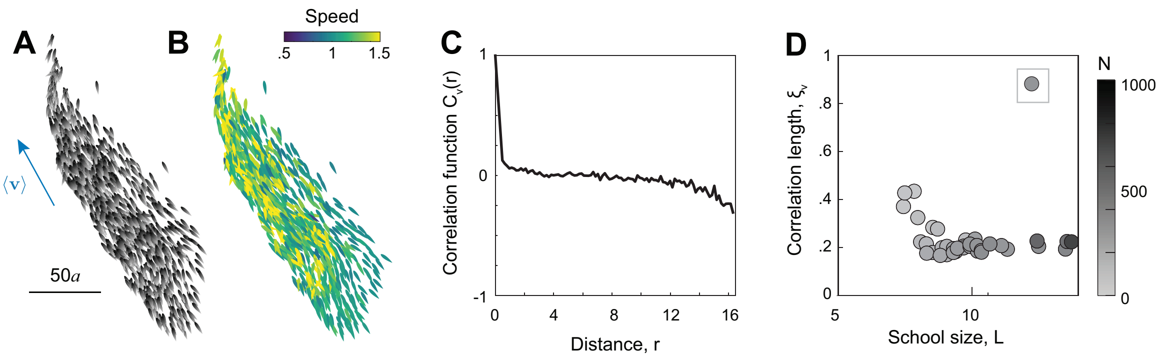

Figure S3: **Spatial correlations in speed and corresponding correlation length. A.** A snapshot of a stable school with $N = 1000$ and **B.** speed of individual swimmers plotted as a colormap. **C.** Correlation function $C_v(r) = \left[\sum_i \sum_j ((||\mathbf{v}_i|| - \langle||\mathbf{v}||\rangle)(||\mathbf{v}_j|| - \langle||\mathbf{v}||\rangle)\delta(r - r_{ij})\right] / C_o \sum_i \sum_j \delta(r - r_{ij})$, where C_o is a normalization constant is the average product of the speed fluctuations of pairs of fishes at mutual distance r . Fitting $C_v(r)$ to an exponential decay $C_v(r) = A\exp(-r/\xi_v)$ gives a fitted correlation length ξ_v. **D.** Correlation length ξ_v plotted versus school size L.

Table S1: **Summary of the dataset generated numerically.** We performed and analyzed 631 distinct simulations at various parameter values and school sizes, each for a total integration time $T = 1000$.

I_a	I_n	I_f	N	ΔN	#MC	#	P
9	0.5	0.01	100	-	5	5	0.96
9	0.5	0.01	1000	-	5	5	0.79
9	0.5	0.01	10,000	-	5	5	0.69
9	0.5	0.01	50,000	-	1	1	0.81
9	0.5	0.01	110-540	10	1	44	0.87-0.96
9	0.5	0.01	550-900	50	1	7	0.78-0.89
9	0.5	0.01	1,500	-	1	1	0.78
9	0.5	0.01	1,600	-	1	1	0.68
9	0.5	0.01	2,000	-	5	5	0.83
9	0.5	0.01	2,500	-	1	1	0.76
9	0.5	0.01	3,000	-	7	7	0.73
9	0.5	0.01	3,600	-	1	1	0.74
9	0.5	0.01	4,900	-	1	1	0.80
9	0.5	0.01	5,000	-	7	7	0.67
9	0.5	0.01	6,400	-	1	1	0.59
9	0.5	0.01	7,500	-	6	6	0.77
9	0.5	0.01	8,100	-	1	1	0.73
9	0.5	10^{-4} – 5	100, 200, 500, 1000	-	5	375	0.67-0.98
9	0.5	10^{-4} – 5	1500, 2000, 2500, 3000	-	1	60	0.66-0.98
9	0.5	10^{-4} – 5	10,000	-	1	15	0.26-0.95
9	0.5	0	100, 1000, 10,000	-	10	30	0.96-0.99
5	0.5	0.01	100-1000	100	1	10	0.83-0.92
5	0.5	0.01	5000	-	1	1	
7	0.5	0.01	100-1000	100	1	10	0.87-0.95
9	0.7	0.01	100-1000	100	1	10	0.80-0.94
9	0.3	0.01	100-1000	100	1	10	0.9-0.97
9	0.3	0.01	5000	-	1	1	
9	0.0	0.01	100, 1000, 10,000	-	1	3	0.92-0.98
9	0.75	0.01	100, 1000, 10,000	-	1	3	0.73-0.87
9	1.0	0.01	100, 1000, 10,000	-	1	3	0.63-0.70

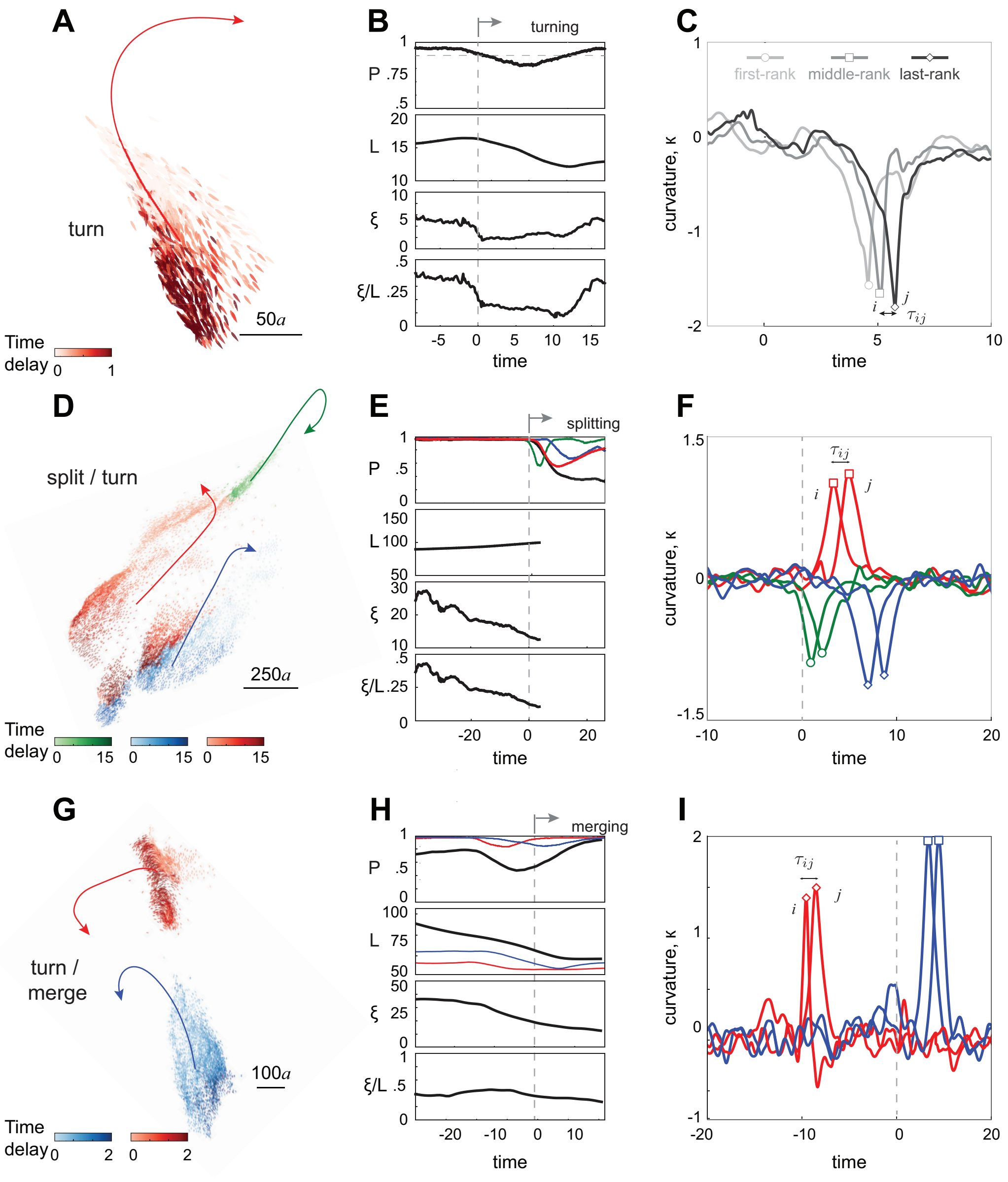

Figure S4: **Analysis of turning, splitting, and merging events. A.** Absolute turning time plotted as a colormap over the fish school at the onset of turning. **B.** Polar order parameter P, school size L, and correlation length ξ, ξ/L versus time. **C.** Sample curvature versus time for first-rank, middle-rank, and last-rank swimmers. (A-C correspond to the turning event in Fig. 5A). **D.** Absolute turning time plotted as a colormap over the fish school prior to splitting. **E.** Polar order parameter, school size, and correlation length for each subgroup versus time. **F.** Sample curvature from each subgroup versus time. (D-F correspond to the splitting event in Fig. 5D). **G.** Absolute turning time plotted as a colormap over the fish school prior to merging. **H.** Polar order parameter, school size and correlation length for each subgroup. **I.** Sample curvature from each subgroup versus time. (G-I correspond to the merging event in Fig. 5G).

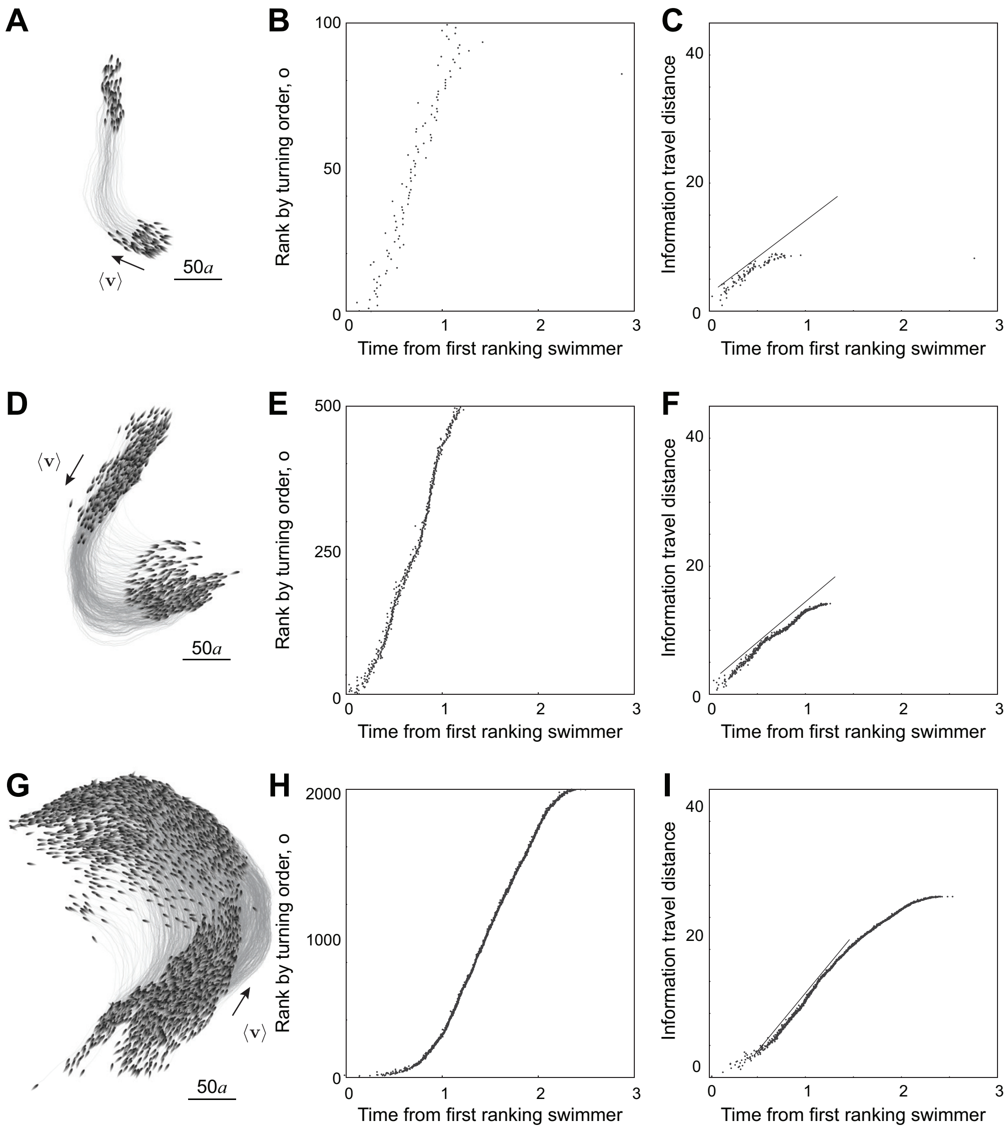

Figure S5: **Analysis of additional turning events.** **A.** Turning trajectories of a school containing 100 fish and **B.** corresponding rank of fish by the order o at which they reach maximal curvature and **C.** information travel distance defined as $\sqrt{o/\text{density}}$ versus absolute turning time delay. The information transfer speed is 11.4 times the individual swimming speed U. **D.** Turning trajectories of a school containing 500 fish and **E.** corresponding rank of fish by the order o at which they reach maximal curvature and **F.** information travel distance defined as $\sqrt{o/\text{density}}$ versus absolute turning time delay. The information transfer speed is 12.7 times the individual swimming speed U. **G.** Turning trajectories of a school containing 2000 fish and **H.** corresponding rank of fish by the order o at which they reach maximal curvature and **I.** information travel distance defined as $\sqrt{o/\text{density}}$ versus absolute turning time delay. The information transfer speed is 18.2 times the individual swimming speed U.

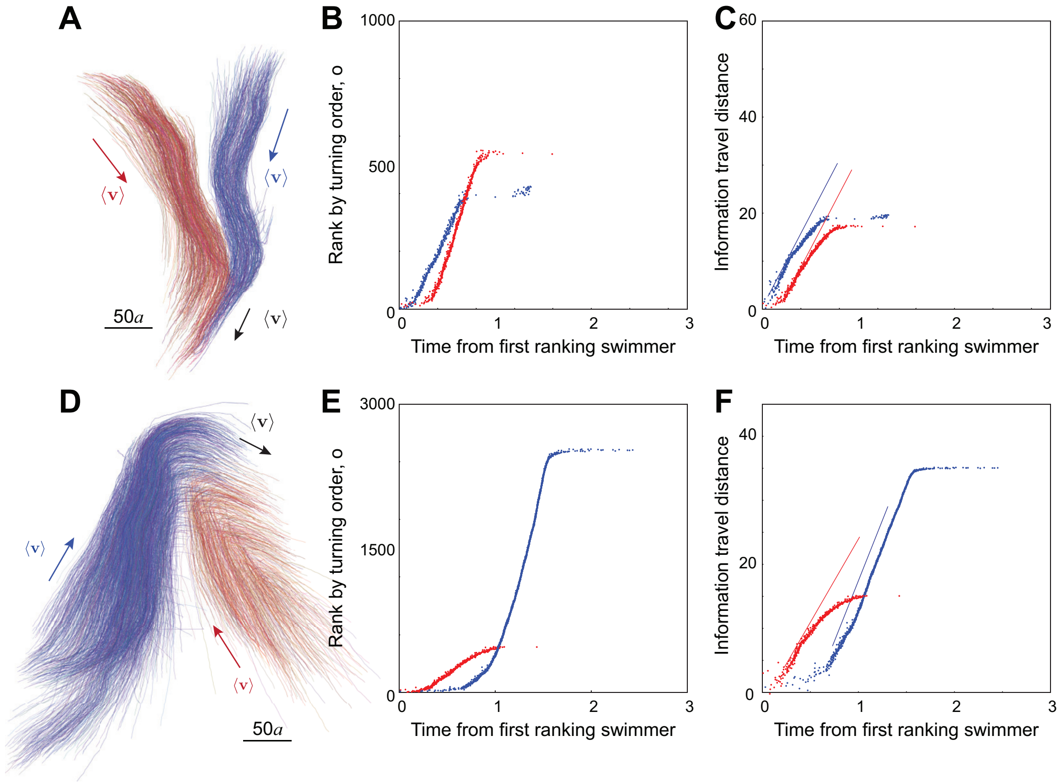

Figure S6: **Analysis of additional merging events. A.** Turning trajectories of a school containing 1000 fish and **B.** corresponding rank of fish by the order o at which they reach maximal curvature and **C.** information travel distance defined as $\sqrt{o/\text{density}}$ versus absolute turning time delay. The information transfer speeds of both clusters are 28.7 times the individual swimming speed U. **D.** Turning trajectories of a school containing 3000 fish and **E.** corresponding rank of fish by the order o at which they reach maximal curvature and **F.** information travel distance defined as $\sqrt{o/\text{density}}$ versus absolute turning time delay. The information transfer speeds of both clusters are 26.0 and 37.8 times the individual swimming speeds U for the red and blue subgroups, respectively.

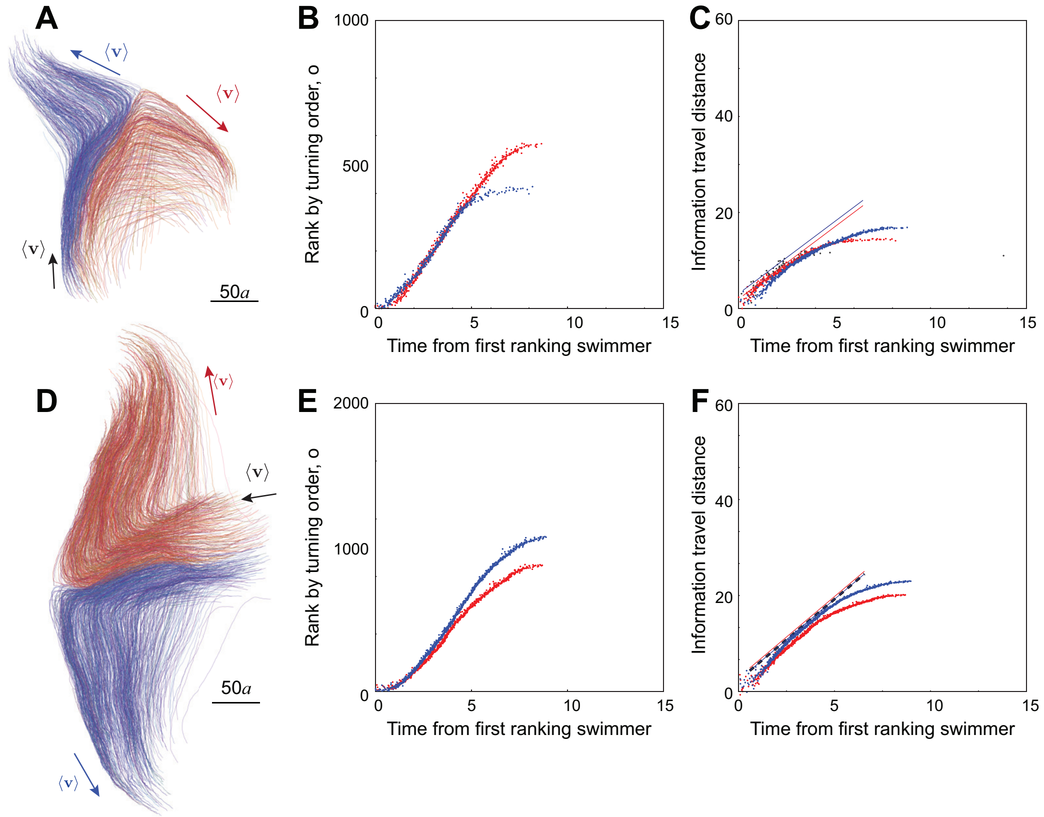

Figure S7: **Analysis of additional splitting events. A.** Turning trajectories of a school containing 1000 fish and **B.** corresponding rank of fish by the order o at which they reach maximal curvature and **C.** information travel distance defined as $\sqrt{o/\text{density}}$ versus absolute turning time delay. The information transfer speeds of both clusters are 3.0 times the individual swimming speed U. **D.** Turning trajectories of a school containing 2000 fish and **E.** corresponding rank of fish by the order o at which they reach maximal curvature and **F.** information travel distance defined as $\sqrt{o/\text{density}}$ versus absolute turning time delay. The information transfer speeds of both clusters are 3.4 times the individual swimming speed U.

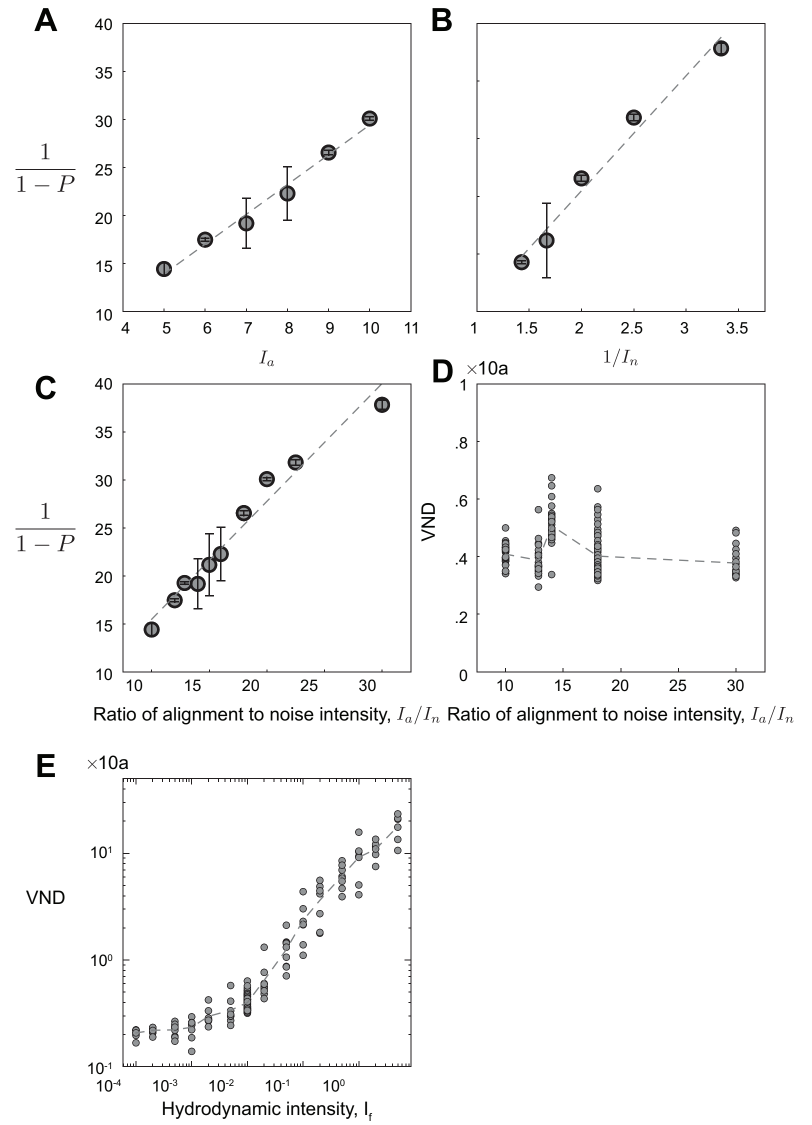

Figure S8: **Scaling with alignment, noise and hydrodynamic intensities.** **A.** Polar order parameter $1/(1-P)$ plotted as a function of alignment intensity I_a. Noise intensity is kept at fixed value $I_n = 0.5$. The fitting curve is $1/(1-P) = 3.11I_a + -1.61$ with $R^2 = 0.98$. **B.** Polar order parameter $1/(1-P)$ plotted versus the inverse of noise intensity $1/I_n$. The fitting curve is $1/(1-P) = 9.99/I_n + 5.49$ with $R^2 = 0.98$. Alignment intensity is kept at fixed value $I_a = 9$. **C.** Polar order parameter $1/(1-P)$ plotted as a function of ratio between alignment intensity and noise intensity I_a/I_n including all simulations from panels A and B. The fitting curve is $1/(1-P) = 1.23I_a/I_n + 3.23$ with $R^2 = 0.964$. **D.** Average distance to Voronoi neighbors as a function of I_a/I_n. In A-D, hydrodynamic $I_f = 0.01$, N =100 to 10,000, $(I_a, I_n) = (9, 0.5)$, $(9, 0.3)$, $(9, 0.7)$, $(5, 0.5)$, $(7, 0.5)$. **E.** Average distance to Voronoi neighbors as a function of hydrodynamic intensity I_f. Parameter values: $I_a = 9$, $I_n = 0.5$. In all panels, five Monte Carlo simulations are performed for each parameter set, each for a total integration time of $T = 1000$.
